# Novel Application of the After-Action Review Technique for Therapeutic Debriefing after Unscheduled Cesarean Births

**DOI:** 10.1055/a-2837-6754

**Published:** 2026-03-30

**Authors:** Elena Lands, Sanjana Ghosh, Anna Binstock, Allison Serra

**Affiliations:** 1 Department of Obstetrics, Gynecology and Reproductive Sciences, Maternal-Fetal Medicine, University of Pittsburgh School of Medicine, Pittsburgh, Pennsylvania, United States; 2 University of Pittsburgh School of Medicine, Pittsburgh, Pennsylvania, United States

**Keywords:** PTSD, trauma-informed care, debrief, quality improvement, patient-derived, patient-centered, birth trauma

## Abstract

**Objective:**

While birth-related posttraumatic stress disorder (PTSD) rates are rising, obstetric providers are ill-equipped to lead the trauma response. To address this need, we employed the After-Action Review (AAR) method in semistructured interviews with patients who recently underwent unanticipated cesarean deliveries. We performed qualitative analyses to determine if the AAR technique could (1) provide a therapeutic outlet to patients who experienced trauma and (2) elicit patient-derived quality improvement opportunities.

**Study Design:**

Twenty patients and their support people were interviewed during the delivery admission, 3 months postpartum, and via an anonymous survey. Two independent coders analyzed transcripts and themes were generated inductively.

**Results:**

All surveyed patients found this process helpful, and 80% suggested an improvement, including educating patients about cesareans earlier and designating a specific staff member to support the patient during a cesarean or code. Five main themes emerged: (1) Mental adaptation to new care plan (reported by 95%), (2) prioritizing safety of baby (85%), (3) external influences on birth expectations (70%), (4) importance of support from various team members (85%), and (5) balance between autonomy and desiring definitive recommendations (75% vs. 30%). Interviewers found the technique easy to apply.

**Conclusion:**

AAR provides a novel, effective, and reproducible mechanism for therapeutic debriefing and generating patient-centered opportunities to improve care within obstetrics.

## Introduction

Within the obstetric population, large-scale surveys 1 and systematic reviews 2 document a high rate of mistreatment and dissatisfaction with care, specifically surrounding unscheduled cesarean deliveries. Unplanned cesarean, especially when performed in an emergency, is an established risk factor for peripartum PTSD and has the potential to be lethal. 3 While these data have led to calls to action 4 and numerous quality interventions designed by health care personnel, 5 generating ideas for quality improvement initiatives directly from patient interviews in the immediate postpartum period has not been described. This study aimed to elicit birthing patients' own suggestions for optimizing their care using an interviewing technique that has not yet been applied to patients.

**Fig. 1:**
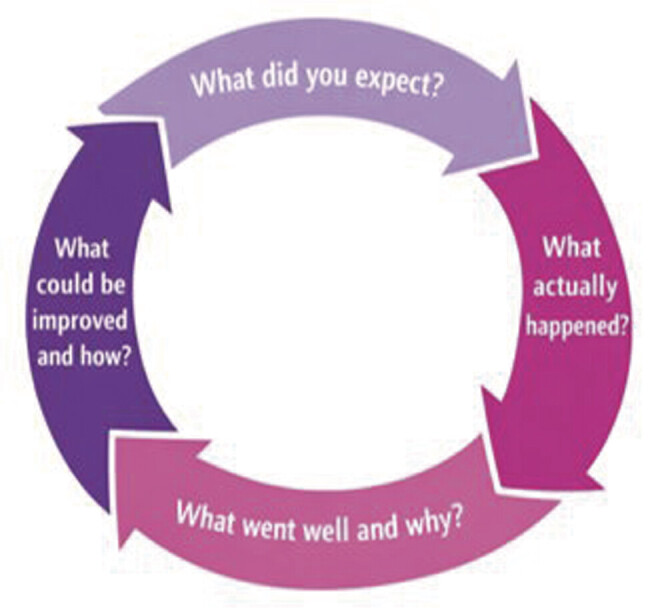
After-action review questions.

After its initial pilot in the 1970s by the U.S. Army, the after-action review (AAR) technique is now used in several industries and public health emergency preparedness systems to devise opportunities for improvement during health crises and other disasters. 6 The World Health Organization (WHO) recommends that health organizations perform these analyses within 3 months of any major health event and has published guidelines on best practices. 7 Briefly, this tool includes four questions: (a) What did you expect to happen? (b) What actually occurred? (c) What went well and why? and (d) What can we improve upon and how? ( Fig. 1 ). 8 The tool provides a replicable framework for critical thinking and allows identification of best practices and performance gaps. While AARs are often used to facilitate organization-level adjustments, we applied the tool during interviews with individual patients who experienced an unscheduled cesarean to (1) characterize patients' perceptions of the health care team's communication surrounding their births, and (2) identify patient-centered improvements to our delivery of labor care.

## Methods

This prospective cohort study included 20 patients who had unscheduled cesareans at a single, tertiary-care academic institution in June 2024. Twenty of these patients' support people also participated in the interviews.

Participants were eligible if they were 18 years or older, English-speaking, and underwent an unscheduled cesarean delivery. Only English-speaking participants were included in this small sample, given the concern for incomplete interpretation of either questions or responses. This could, in turn, lead to misinterpretation of the themes of their responses by the English-speaking coders. Patients were approached on postoperative day 1, 2, or 3 by their care team rather than the research team. Written informed consent was obtained by the study team from interested participants. Consenting participants were interviewed while still admitted to the hospital, were subsequently contacted 3 months postpartum by phone, and finally were administered an anonymous online survey.

Interviewers were trained in the AAR technique and were proctored prior to completing study conversations. All patients were interviewed with the same script, including the basic four questions of the AAR technique: (a) What did you expect your birth to be like? (b) What actually happened? (c) What went well and why? and (d) What can we improve and how? Additionally, all patients were asked what they would recommend offering to patients after their birth and what they would have found helpful. Prompts were used after each of these questions to facilitate a deeper understanding of the influences of various sources of information on their expectations, and of different health care team actors on their experiences.

Audio recordings were transcribed into documents that were de-identified and uploaded to the NVivo platform. 9 Two independent coders analyzed interview transcripts and themes were generated inductively through close reading. 10 A preliminary codebook was made after analysis of the first three transcripts, then iteratively modified after subsequent transcripts were reviewed. Individual codes were grouped into shared themes using constant comparison analysis as well as into categories based on which types of providers were mentioned in their comments. Each transcript was discussed until consensus on recurrent themes was reached. 11 Support people's experiences and corresponding themes were analyzed separately from patients' accounts. Interviews were completed until thematic saturation was reached and no new themes were identified. 12

Patients' demographic and clinical information were collected via chart review and were analyzed after all qualitative analyses were completed. Institutional Review Board approval was obtained.

## Results

Eighty-four percent of patients who were approached consented. Of the 20-patient cohort, 20% identified as non-White and 20% were publicly insured. Thirty-five percent of neonates were admitted to the neonatal intensive care unit (NICU), and 15% were preterm.

From the qualitative analyses, five main themes emerged: (1) Mental adaptation to a new care plan, (2) prioritizing the safety of the baby, (3) the importance of support from various team members, (4) external influences on birth expectations, and (5) the balance between desiring autonomy versus definitive recommendations, with 40% of patients affirming conviction in their decision to proceed with cesarean ( Table 1 ). All but one participant reported a shift from expectation to reality, requiring the need to mentally reorient. Eighty-five percent focused on the baby's outcome, sometimes at the expense of their own well-being or understanding. Eighty-five percent identified specific individuals (nurse, anesthesia provider, obstetrical provider, doula, social worker) whose presence or absence made a big difference. Seventy percent described how cultural influences, including social media, friends, or family, colored their expectations of birth. Finally, while 75% of patients appreciated the opportunity to ask questions and were empowered to make their own decision, 30% requested a definitive recommendation (“I asked the doctor, if it would be your baby, would you do a C-section?”).

**Table 1:** Themes and quotations of the patient experience of having an unplanned cesarean birth

Theme	Subthemes (patients [percentage], number of references)	Example
External influences on birth expectations	Glamorizing birth (7 [35%], 11)	“... you hear of how a birthing process is supposed to take place... and then you get to that position, and it doesn't work for your body.”
	Family (5 [25%], 9)	“Everything that I knew about a C-section going into my own was what I had heard from my own mom.”
	Peers' birth experiences (4 [20%], 10)	“A lot of my friends who have been induced had failed inductions.”
	Media or educational resources (3 [15%], 4)	“Being a woman and learning in health class about the birth process... I kind of had it in my mind that birth was going to be a specific way.”
Prioritizing baby	Prioritizing the safety of the baby (17 [85%], 42)	**“... I'd rather have a c-section than a dead baby.”**
	Missing infant bonding (13 [65%], 25)	“I like barely even got to see what my baby looked like.”
Balance between patient autonomy and desiring definitive recommendations	Conviction in their decision for cesarean (8 [40%], 16)	“It was the best decision we made; it was the right decision and [my baby]'s safe and good and so am I.”
	Desiring definitive recommendation (6 [30%], 12)	“I asked [the doctor], if it would be your baby, would you go to C-section? And she said yes.”
	Felt team did all they could (6 [30%], 8)	“I don't feel there was really anything that they could have done to make my body or make her go a different way.”
Adaptation to the new plan of care	Quick transition in plan of care (14 [70%], 27)	“One of the most memorably drastic things for me was going from the dark, quiet room where I was almost asleep to the most, bright lights and commotion, sort of all at once.”
	Volume of personnel (10 [50%], 24)	“... it was very hectic, like these thousands of people running around and telling stuff.”
Importance of support from the health care team members (overall, 20 [100%], 84)	Support from the nurse (11 [55%], 21)	“I was bawling with like snot dripping down my face and the LDR nurse, her name was [redacted], was wiping the snot off my face... She was like the most amazing human ever.”
	Support from OB providers (9 [45%], 14)	“The doctor introduced himself that he would do the C-section... he was very calm, and I felt like OK, it looks like I'm in good hands.”
	Support from a doula (5 [25%], 9)	“[My doula] literally stood by my side the whole time, so I appreciate that.”
	Support from anesthesia (4 [20%], 4)	“The anesthesia team was very helpful... she always was like bending down [to ask] like, what do you need?”
	Importance of familiarity with the provider (3 [15%], 5)	“I feel like when someone knows you, you feel like they're more invested in your care.”
	**Lack** of awareness and support (7 [35%], 11)	“The next thing I remember was [the doctors] saying ‘she needs to stop throwing up, her bowels are coming out.’ Uh, so that was super fun.”
	Dissatisfaction with learners/trainees (8 [40%], 8)	“... [a resident] is putting a needle in my back and he asked for another doctor to come help and said, ‘I think I’m hitting the bone.' **We just need to remember to treat people like humans and not like a patient to work on.** ”
Strong emotional reactions	Fear (16 [80%], 30)	“I was in a full like panic attack for those 10 minutes.”
	“Traumatizing” (6 [30%], 9)	**“This was probably one of the most traumatic things that I've had to endure or go through to bring her into this world.”**
	Disappointment in self (6 [30%], 14)	“I was like, ‘did I not try hard enough?’ ‘What did I do wrong?’”
	Postpartum recovery harder than expected (10 [50%], 18)	“I feel a lot of pain with the C-section and even the first 2 days it was like I can't even move off the bed...”
Views on AAR project	Helpful (30% initially, 100% surveyed later)	“Having a space to talk about my unplanned birth experience shortly after it happened was extremely helpful. I am still processing my birth, but I believe that being able to reflect on the experience early on during the interview provided the first steps for processing.”

Abbreviation: AAR, After-Action Review.

Seventy percent of patients reported instances of receiving positive communication from care team members, highlighting the importance of early communication about the possibility of cesarean and acknowledgment of their emotions during their delivery course ( Table 2 ). In contrast, 55% reported instances of negative communication, with one stating, “We just need to remember to treat people like humans and not like a patient to work on.” Overall, 30% used the word “trauma” to describe their experience, and 65% reported missing infant bonding opportunities. While patients reported more positive than negative communication, partners had the reverse experience, with 40% noting negative versus 20% positive instances.

**Table 2:** Quality of communication from care team

	Positive (patients (percentage), references)	Negative (patients (percentage), references)
Communication to the patient	13 (65%), 22	11 (55%), 29
Communication to the partner/support people	4 (20%), 4	8 (40%), 15
Perceived communication within the health care team	2 (10%), 3	2 (10%), 5
Pacing of decision-making	5 (25%), 10	8 (40%), 10

All surveyed patients indicated they found the interview process helpful, with several praising the opportunity to “express myself with no judgment,” and that “it really felt like therapy.” Another stated, “Having a space to talk about my unplanned birth experience shortly after it happened was extremely helpful… I believe that being able to reflect on the experience early on during the interview provided the first steps for processing.”

In addition to the common themes extracted, 80% of patients suggested improvements in the workflow preceding and during an unplanned cesarean. Suggestions included educating the patient about the possibility of cesarean (25% suggested this with two patients recommending this occur during the delivery admission and five recommending it prenatally) and designating a staff member to support the patient during a cesarean or code event (20%). Similar suggestions were made during the subsequent phone interview 3 months later, with no new themes identified. Several patients appreciated the opportunity to offer feedback. One participant stated that “…going through the patients [directly] is a wonderful idea to really get good, true feedback that you can hopefully take to try to solve things.”

Finally, interviewers found that the technique allowed conversation to flow naturally and thought it was easy to remember without requiring notes.

## Discussion

The purpose of this study was to evaluate how effectively the AAR method captures insights from individuals' experiences after unplanned cesarean deliveries. Both patients and interviewers universally described the method as helpful. While some themes were broadly expressed, we uncovered a spectrum of perspectives on the optimal method of delivering information. This underscores the importance of educating providers about altering their communication style for each individual they treat, and to provide trauma-informed care whenever possible. Subsequent analysis revealed that communication to support people offered a specific opportunity for improvement, as negative instances were described more often than positive instances.

Our secondary aim addressed the need for patient-centered innovations to deliver improved peripartum care. Patients communicated their desire to contribute to quality improvement, and a majority offered useful recommendations. The recurrent suggestions for assigning a designated support person during code events, ensuring provider continuity, and initiating earlier education on cesarean delivery reinforce the validity of these interventions. These data are being applied within our health system to develop training materials for obstetrical providers as well as educational communication to patients to address the educational gaps identified.

The benefits of AAR for teams have been well-described and include creating a sense of ownership to facilitate corrective actions being taken, providing clear documentation, and building consensus for future events. AAR has been shown to foster interprofessional and intercultural learning, and this study demonstrates that it can also foster learning between patients and providers.

This study is unique in its application of an established framework to debrief individual patients, as well as its integration of interviews at two time points. Despite the range of racial and ethnic identities and insurance statuses represented, our results may not be entirely generalizable to every care center, given that this study was conducted at a single academic institution. However, this study demonstrates that this method is easily replicated in new settings, from the battlefield to a postpartum care unit.

## Future Directions

Training providers to conduct AARs enhances their ability to both offer therapeutic support to patients experiencing unexpected outcomes and to uncover innovative opportunities for quality improvement. This technique may be applied to generate patient-derived and patient-centered initiatives in several fields of medicine.
